# Data Visualization Support for Tumor Boards and Clinical Oncology: Protocol for a Scoping Review

**DOI:** 10.2196/53627

**Published:** 2024-03-05

**Authors:** Dominik Boehm, Cosima Strantz, Jan Christoph, Hauke Busch, Thomas Ganslandt, Philipp Unberath

**Affiliations:** 1 Medical Center for Information and Communication Technology Universitätsklinikum Erlangen Friedrich-Alexander-Universität Erlangen-Nürnberg Erlangen Germany; 2 Bavarian Cancer Research Center (Bayerisches Zentrum für Krebsforschung) Erlangen Germany; 3 Chair of Medical Informatics Friedrich-Alexander-Universität Erlangen-Nürnberg Erlangen Germany; 4 Junior Research Group (Bio-)medical Data Science Faculty of Medicine Martin-Luther-University Halle-Wittenberg Halle Germany; 5 Group for Medical Systems Biology Lübeck Institute of Experimental Dermatology University of Lübeck Lübeck Germany; 6 SRH Fürth University of Applied Sciences Fürth Germany

**Keywords:** clinical oncology, tumor board, cancer conference, multidisciplinary, visualization, software, tool, scoping review, tumor, malignant, benign, data sets, oncology, interactive visualization, data, patient, patients, physicians, medical practitioners, medical practitioner, conference

## Abstract

**Background:**

Complex and expanding data sets in clinical oncology applications require flexible and interactive visualization of patient data to provide the maximum amount of information to physicians and other medical practitioners. Interdisciplinary tumor conferences in particular profit from customized tools to integrate, link, and visualize relevant data from all professions involved.

**Objective:**

The scoping review proposed in this protocol aims to identify and present currently available data visualization tools for tumor boards and related areas. The objective of the review will be to provide not only an overview of digital tools currently used in tumor board settings, but also the data included, the respective visualization solutions, and their integration into hospital processes.

**Methods:**

The planned scoping review process is based on the Arksey and O’Malley scoping study framework. The following electronic databases will be searched for articles published in English: PubMed, Web of Knowledge, and SCOPUS. Eligible articles will first undergo a deduplication step, followed by the screening of titles and abstracts. Second, a full-text screening will be used to reach the final decision about article selection. At least 2 reviewers will independently screen titles, abstracts, and full-text reports. Conflicting inclusion decisions will be resolved by a third reviewer. The remaining literature will be analyzed using a data extraction template proposed in this protocol. The template includes a variety of meta information as well as specific questions aiming to answer the research question: “What are the key features of data visualization solutions used in molecular and organ tumor boards, and how are these elements integrated and used within the clinical setting?” The findings will be compiled, charted, and presented as specified in the scoping study framework. Data for included tools may be supplemented with additional manual literature searches. The entire review process will be documented in alignment with the PRISMA-ScR (Preferred Reporting Items for Systematic Reviews and Meta-Analyses extension for Scoping Reviews) flowchart.

**Results:**

The results of this scoping review will be reported per the expanded PRISMA-ScR guidelines. A preliminary search using PubMed, Web of Knowledge, and Scopus resulted in 1320 articles after deduplication that will be included in the further review process. We expect the results to be published during the second quarter of 2024.

**Conclusions:**

Visualization is a key process in leveraging a data set’s potentially available information and enabling its use in an interdisciplinary setting. The scoping review described in this protocol aims to present the status quo of visualization solutions for tumor board and clinical oncology applications and their integration into hospital processes.

**International Registered Report Identifier (IRRID):**

DERR1-10.2196/53627

## Introduction

Multidisciplinary team interventions, and especially tumor boards (or multidisciplinary cancer conferences [MCCs]) have been shown to significantly improve the quality of cancer care [1-3]. Complex, multimodal, and ever-growing data sets in multidisciplinary settings present special challenges when it comes to data visualization [4]. These data sets can include anything from demographic information and laboratory results to tumor imaging, pharmacotherapeutic timelines, and genomics data, providing limitless opportunities for aggregation and joint visualization. The need for digital support and customized visualization solutions becomes especially apparent when discussing the time constraints often found in clinical oncology settings. Available time frames for treatment decisions might range between 10 and 20 minutes per patient [5], highlighting the importance of aggregated and annotated data to enable participating health care professionals to include all relevant information in the decision-making process. However, even without taking limited time into account, the growing complexity of patient data makes it difficult to fully understand a patient’s health status without the support of visualization, even more so when multiple points of data on the patient journey are available [6,7]. While tools for the visualization of multimodal data in the described settings exist [8-10], there is no current overview of actively used and established visualization tools and their key differences, especially on an international level.

The rise of molecular tumor boards (MTBs) has been an additional driving factor in the development of digital support applications for interdisciplinary settings and the incorporation of multimodal data. Combining ordinary clinical information with the complexity of genomics data required special tooling to enable oncologists to make fully informed treatment decisions and limit the time necessary for the MTBs’ preparation [11,12]. Virtual MTBs and rising numbers of outpatient referrals lead to a heterogeneous pool of tumor board participants and increase the need for intuitive visualization. Complex patient journeys have to be presented in a condensed and clear manner, sometimes crossing language barriers, without prior knowledge of the patient or their history [12,13]. This led to the emergence of software solutions such as cBioPortal (Memorial Sloan Kettering Cancer Center) [14-16], The Cancer Core Europe Molecular Tumor Board Portal [17], and AMBAR [18], offering complex visualization of genomic variants found in cancer samples.

Additionally, knowledge bases such as OncoKB [19] or CIViC [20] have been created, providing access to annotations with aggregated and structured information on available targeted therapies. The usage of these established tools not only supports the preparation and execution of MTBs but also increases the consistency of therapy recommendations between molecular tumor conferences, even for patients with rare cancers and mutation patterns [21]. Research indicates that differing processes and tools may lead to inconsistent therapy recommendations [22].

To enable the visualization of data for the preparation and execution of MCCs, patient and supplementary data must be made available to the tools used. This is challenging as the availability of interfaces or APIs for the import of data sets can vary greatly and is often only possible through the use of proprietary data formats, which may require the development of extract, transform, and load processes to automate data import and export [23]. Additionally, data privacy regulations and ethical concerns may limit the usage of external services. Several German initiatives and consortiums (eg, Medical Informatic Initiative [24], Bavarian Center for Cancer Research [25], and German Network for Personalized Medicine [26]) are working on standardized data sets and processes related to the cancer patient journey in German hospitals. This includes software extensions for established tools such as cBioPortal, for example, covering the documentation and visualization of therapy decisions in MTBs [27,28].

In summary, there is an increasing need for additional visualization in the context of tumor board settings to leverage the full potential of growing data sets for patient care and therapy decisions. Integrating these software solutions into clinical processes is a challenging task, requiring data from a variety of sources to be readily available to facilitate their use in the preparation and execution of tumor boards. With this in mind, the objective of the proposed scoping review is to identify available software support for MCCs described in scientific literature, gather key aspects of applied visualization strategies as well as their integration into existing processes, and present them in a comprehensive overview.

## Methods

### Design

For conducting this scoping review study, we will use the scoping study framework of Arksey and O’Malley [29] as a methodological blueprint for this review.

Arksey and O’Malley describe a five-stage model for scoping study design: (1) identification of the research questions; (2) identification of relevant studies; (3) study selection; (4) data extraction and charting; and (5) collating, summarizing, and reporting the results. Any subsequent deviations of the final report from the scoping review protocol will be highlighted and explained in the scoping review report.

### Stage 1: Identification of the Research Questions

While there has been a continuous development of digital support tools for clinical oncology settings over the last few years, currently no structured overview of the visualization tools and techniques used in these applications exists. The core research question driving the scoping review was proposed based on these circumstances, and further developed through multiple iterations of discussion in the research team:

Research question: What are the key features of data visualization solutions used in molecular and organ tumor boards, and how are these elements integrated and used within the clinical setting?

Starting from this overarching research question, specific questions we wanted to answer while extracting data from relevant literature were developed. These were used at a later step to design the data extraction template. They were the following: (1) What data visualization solution is being used? (2) What kind of data are being visualized? (3) How do they visualize the available data? (4) How are these elements integrated and used within the clinical setting? (5) How accessible are the solutions? (6) Are the solutions already being used in hospitals? (7) Have the proposed or implemented solutions been evaluated?

### Stage 2: Identification of Relevant Studies

#### Core Concepts and Keywords

To find relevant studies and gain an insight into the search domain an initial manual literature search was executed. In total, 19 key papers were identified and used to extract concepts for the development of a search strategy. All in all, we were able to identify 3 core concepts that relevant literature would have to encompass. First, tumor boards or similar settings as the target domain. Second, software or some other form of digitalized support. Lastly, the described mode of support delivered by the software, for example, visualization, usage as a decision support system, or personalized medicine.

In the following process, these concepts were used to define keywords for the development of individual search strategies for the chosen databases (Textbox 1). The initial manual literature search showed that relevant literature can be found in a variety of different contexts. Supporting applications for tumor boards may be described in publications covering the development of those tools, their integration into hospital processes, evaluations of their efficacy, or even as a sidenote in the medical literature. As such some of the keywords may seem out of scope at first glance but lead to the inclusion of additional relevant results. MTBs present one of the driving institutions for the development of multimodal and interactive visualization solutions for clinical oncology settings and as such provide a variety of keywords to the search strategy.

**Concepts and corresponding keywords.**

**Target domain**

- Tumor board, tumor conference, molecular tumor board, mutation database, and cancer genomics.

**Software**

- Virtual, digital, software, tool, platform, and portal.

**Mode of support**

- Visualization, interactive, preparation, usability, clinical decision support system, personalized medicine, and precision medicine.

#### Query Construction

Using the defined concepts and corresponding keywords, search strategies for the different databases were built. The general strategy for this process was connecting concepts through “AND” operators, while keywords were connected by “OR” operators. The search was limited to title and abstract where possible since this proved to consistently recall key literature and articles of interest while greatly reducing the amount of out-of-scope search results. The queries were adapted to the specific database needs, for example, the usage of Medical Subject Heading Terms for PubMed. The resulting queries were tested on their recall of key literature and accuracy. They underwent an iterative optimization process based on their performance and in a last step were validated by a librarian. The proposed queries can be found in Multimedia Appendix 1. All future deviations will be documented and discussed in the final publication.

### Stage 3: Study Selection

#### Inclusion and Exclusion Criteria

Only articles published in English during the last 10 years will be included. Since our initial search showed a very broad range of target literature types, we decided not to use further inclusion or exclusion criteria to include all potentially relevant articles.

#### Selection Process

All literature found by applying the search strategies to PubMed [30], Web of Knowledge [31], and SCOPUS [32] will be exported into a compatible format and uploaded to Rayyan (Rayyan) [33], which will be used for the 2-step study selection process. In the first step, title-abstract screening will be performed to quickly exclude out-of-scope literature, reducing the workload for the full-text screening stage. Each paper will be screened by at least 2 reviewers. An additional reviewer will solve conflicting inclusion decisions. During the second screening phase, full-text screening will be performed to exclude results that will not assist in answering the research question described in Stage 1. To increase consistency, criteria for the inclusion and exclusion of literature during the screening process will be supplied to all participating researchers and discussed in a meeting before the start of the screening. The study selection process will be documented using Rayyan. The results of this process will be compiled into a PRISMA-ScR (Preferred Reporting Items for Systematic Reviews and Meta-Analyses extension for Scoping Reviews) [34] flowchart.

### Stage 4: Data Extraction and Charting

All selected literature will be searched for metadata and information relevant to answering the proposed research question. For a standardized approach between reviewing parties, a data extraction template (Table 1) was developed, encompassing all required metadata, as well as specifics regarding each aspect of the overarching research question. The data extraction template was designed in a close fashion to templates from similar review projects [35,36] as their authors were consulted during the development process of this protocol. Potential modifications to the template will be documented, properly highlighted, and discussed in the final publication.

**Table 1:** The data extraction tool template.

Data and item		Description
**Metadata**		
	Title^a^	Title
	Citation details^a^	Author (first), journal, and DOI
	Year of publication^a^	Year of publication
	Publication type^a^	Type of publication
	Institute^a^	Corresponding institute
	Funding source	Funding source of the publication
	Objective^a^	Publication objective
	Methods^a^	Short description of the methodology used
	Summary result^a^	Short description of the results
	Conclusion	Short description of the conclusion
	Keywords	Keywords
	Citations	List of articles citing this paper
**Results related to the research question**		
	What data visualization solutions are being used?	Name and description of the applications or tools that are being used
	What kind of data are being visualized?	List and description of the data that is being visualized. Name and a short description of the corresponding standardized or harmonized data set if applicable
	How do they visualize the available data?	List and description of the modes of visualization applied, including interactive features such as filtering or customization
	How are these elements integrated and used within the clinical setting?	Description of the integration into the clinical setting if available, covering the process integration as well as software interfaces and capabilities for the documentation of tumor boards, if available
	How accessible are the solutions?	Accessibility of the software, for example, open source or commercial product, and licensing information
	Are the solutions already being used in hospitals?	List of hospitals that are already using the software
	Has there been a methodical evaluation of the proposed or implemented solution?	Description of the evaluation methodology and the corresponding results, if available

^a^Mandatory field.

### Stage 5: Collating, Summarizing, and Reporting the Results

After data extraction and charting, the results will be analyzed in a 2-step process per Arksey and O’Malley’s framework [29]. First, the findings will be analyzed numerically, comparing the extent, nature, and distribution of the literature found. Following that we will prepare a thematic overview on visualization solutions for tumor boards and clinical oncology. The visualized data per tool, as well as the respective visualization strategies used and remaining elements of the data extraction template, will be charted and appropriate graphics will be created. The findings will be presented following the PRISMA-ScR reporting guidelines [34].

### Ethical Considerations

Since our review will not involve human participants, this study does not require ethics approval.

## Results

The scoping review started with a tentative search beginning in September 2023 leading to 2057 results with a suspected 1227 duplicates. In the next step titles and abstracts will be iteratively screened by reviewers to decide on the paper’s inclusion in the further review process (see stage 3). This will be based on the criteria described in the Methods section. Included articles will be analyzed by applying the appended data extraction tool (see stage 4). This step is expected to be finished by December 2023. Lastly, the results will be summarized and compiled (see stage 5) up until the beginning of 2024. We expect them to be published during the second quarter of 2024.

## Discussion

We designed a scoping review, aiming to present the current state of software support for clinical oncology settings, focusing on visualization solutions used in MCCs and their integration into hospital processes. The initial search, executed using the methods and queries described in this protocol, was able to show that a significant amount of potentially relevant literature can be found in the selected electronic databases. By asserting that manually identified key papers are included in the results, we are confident that the results produced by the search queries include the target domains.

However, a potential limitation of the completeness of the scoping review might be its focus on scientific literature. While tumor boards are often actively part of research projects and publicize their findings including used software solutions, commercial visualization tools and supporting software are being used as well. These might present new and interesting approaches but would not be necessarily found during a literature search. However, limited access to these applications would make their analysis difficult, and closed-source solutions often present limited possibilities for extensions and follow-up work. Additionally, available literature on supporting software might focus on features apart from visualization and offer limited insight into the questions posed. We aim to mitigate this by supplementing the findings through additional manual searches for included software.

We expect the scoping review’s findings to show the current state of data visualization in clinical oncology settings. By comparing these results with the data most commonly available in the context of tumor boards and upcoming data sets we aim to anticipate visualization needs and provide starting points for more focused requirement analysis. Lastly, we hope to inform the future development of flexible visualization solutions for expanding oncology data sets.

## Appendix

Multimedia Appendix 1 Proposed search queries for PubMed, Web of Knowledge, and SCOPUS.

## Abbreviations

MCC: multidisciplinary cancer conference
MTB: molecular tumor board
PRISMA-ScR: Preferred Reporting Items for Systematic Reviews and Meta-Analyses Extension for Scoping Reviews
