# Supplementary material for: Data Visualization Support for Tumor Boards and Clinical Oncology: Protocol for a Scoping Review
Source: JMIR Res Protoc. 2024 Mar 5;13:e53627. doi: 10.2196/53627 (PMC10951826; doi:10.2196/53627)
Supplement: Multimedia Appendix 1 [file resprot_v13i1e53627_app1.docx]

## **Multimedia Appendix 1.** Data visualization support for tumor boards and clinical oncology: search strategy.

| Web Of Knowledge | TS=(((tumor board OR tumor conference OR MTB OR "mutation database" OR "cancer genomics") AND (virtual OR digital OR tool OR software OR platform OR portal)) AND (visual* OR interactive OR preparation OR usability OR "clinical decision support system" OR "personalized medicine" OR "precision medicine")) |  |
| --- | --- | --- |
| Scopus | TITLE-ABS-KEY ( ( tumor AND ( board* OR conference* ) ) OR "MTB" OR "mutation database" OR "cancer genomics") AND TITLE-ABS-KEY ( virtual OR digital OR tool OR software OR portal OR platform) AND TITLE-ABS-KEY ( visual* OR interactive OR preparation OR usability OR "clinical decision support system" OR "personalized medicine" OR "precision medicine") |  |
| PubMed | (((tumor board*[tiab] OR tumor conference[tiab] OR MTB[tiab] OR mutation database[tiab] OR cancer genomics[tiab]) AND (virtual[tiab] OR digital[tiab] OR tool[tiab] OR software[tiab] OR platform[tiab] OR portal[tiab])) AND (visual*[tiab] OR interactive[tiab] OR preparation[tiab] OR usability[tiab] OR clinical decision support system [MeSh Terms] OR personalized medicine[MeSh Terms] OR precision medicine[tiab])) |  |
